# Indoxacarb poisoning: An unusual presentation as methemoglobinemia

**DOI:** 10.4103/0972-5229.45082

**Published:** 2008

**Authors:** Lakshmi Prasanna, S. Manimala Rao, Vishal Singh, Rash Kujur, Gowrishankar

**Affiliations:** **From:** Department of Anaesthesiology and Critical Care, Yashoda Hospitals, Somajiguda, Hyderabad, India

**Keywords:** Indoxacarb, methemoglobinemia

## Abstract

We describe the management of a case of methemoglobinemia secondary to ingestion of indoxacarb, an oxadiazine insecticide.

## Introduction

Indoxacarb is an oxadiazine insecticide, which acts by blocking sodium channels in the nervous system of insects and causes mild tremors, cessation of feeding, and death in a couple of hours. Contact with the substance can take place through ingestion, physical contact, translaminar action, during preening, and at rewetting of surfaces.[1]

In our patient methemoglobinemia occurred following ingestion of indoxacarb in a suicidal attempt. This case report highlights the importance of considering the possibility of methemoglobinemia in cases of exposure to indoxacarb and its early recognition and management.

## Case Report

A 48-year-old male farmer came to the emergency department in the early hours of the day, with a history of consumption of unknown poison (suicidal attempt) followed by vomiting. He had received gastric lavage and atropine at a local hospital. On examination, his Glasgow coma scale score was 13/15 and afebrile. His vital signs were stable. He was shifted to Acute Medical Care Unit (AMCU) with oxygen supplementation.

On examination in AMCU, the patient was drowsy and hypoventilating, his pupils were bilaterally mid-dilated and reacting to light, His SpO_2_ was 86% on 6 lit / min of oxygen at Therefore, a decision was taken to intubate the patient and commence mechanical ventilation and commence mechanical ventilation. Organophosphorus poisoning was suspected and Atropine 0.2 mg/hr and PAM-200 mg/hr infusions were started. Routine and special investigations were carried out and the results were : S.Cholinesterase-0.76 KU/L (0.6-1.5), blood for barbiturates was negative, stomach wash for barbiturate and benzodiazepines negative, LFT showed SGOT of 128 IU/L, SGPT of 109 IU/L; S. Creatinine was normal, Urine-pH-5.8, Specific gravity-1.016, no pus cells, no RBC, TLC-18,600.

On the same day in the evening, the patient's blood was noticed to be muddy brown in colour, and peripheral cyanosis (bluish brown) was noticed. An arterial blood gas analysis revealed a paO_2_ of 347 mmHgon a FiO_2_ of 0.6 and the SpO_2_ was only 86%. The patient's relatives were thoroughly questioned again regarding the poison and they brought the tin from which the patient consumed the poison. It was examined and found to be INDOXACARB, a non-organophoshorous oxadiazine insecticide. PAM and atropine were stopped. After ruling out other poisons, and with a high degree of suspicion, Methemoglobinemia was considered which could be secondary to ingestion of indoxacarb. Blood was sent for methemoglobin level and co-oximetry. Methemoglobin level was found to be 33.4%. Immediately sterile inj. Methylene blue in 100 ml normal saline, was administered intravenously at a rate of 1mg /kg.[2] Inj.Vitamin-C 1g IV along with dextrose containing fluids. Methylene blue 60 mg IV BD and Inj. Vitamin-C 1 g IV were continued. The patient gradually started showing signs of improvement and his SpO_2_ gradually improved to 92 - 95% on day two. The patient was gradually weaned off ventilatory support. The SpO_2_ increased to 95% on day three and to 95-97% on day four. The patient was subsequently extubated on day four and continued to maintain a good gas exchange on face mask oxygen. On day five, the SpO_2_ was 98% on face mask with 5 liters/min. of oxygen. On day six, the methemoglobin level was 2%. The patient was discharged from ICU on day seven.

Thus our patient was managed successfully with methylene blue and other supportive/symptomatic treatment for methemoglobinemia which was probably secondary to indoxacarb poisoning.

## Discussion

Indoxacarb is an oxadiazine pesticide used for control of cotton bollworm and native budworm in cotton and soyabeans.[3] Our patient had consumed indoxacarb in a suicidal attempt. In the above mentioned case, our patient had developed peripheral cyanosis and had muddy brown discoloration of blood. With an index of suspicion, after ruling out other poisonings, methemoglobinemia was diagnosed and treated. Methemoglobin is generated by oxidation of the heme iron moieties to ferric state, causes bluish-brown muddy color resembling cyanosis. It has got very high affinity to oxygen and oxygen is not delivered to the tissues (oxygen dissociation curve shifted to the left). It is suspected in patients with hypoxic symptoms who appear cyanotic but have a sufficiently high PaO_2_. Muddy appearance of freshly drawn blood is a critical clue for diagnosis.[2] Normal Meth-Hb levels are <1%. The physiologic reduction of MethHb Fe3+ to Hb Fe_2_+ is mainly accomplished by red cell NADH-cytochrome b5 reductase.

It is manifested by Muddy or Chocolate brown colored fresh blood, Bluish discoloration of skin and mucosa (at 1.5g% or 10% of meth-hb), irritability, seizures. Cerebral ischemia occurs at >15% and >60% are lethal.[2]

It can be congenital or acquired due to drugs/chemicals. It can be diagnosed by co-oximetry,[4] methemoglobin levels,[4] ABG, SpO_2_. In our patient methemoglobin was 33.4% and the patient had all the classical clinical features.

Treatment includes ceasing the offending agent, correcting the metabolic abnormalities, administering methylene blue at a dose of 1-2 mg/kg loading dose q 30-60 min, to a maximum of 7 mg/kg, followed by 50-100 mg twice or thrice daily[4] and other supportive measures as required. In our patient, we instituted treatment with 1 mg/kg of methylene blue as a loading dose, followed by 60 mg twice daily. Methylene blue gets reduced to leucomethylene blue which in turn reduces methemoglobin by NADPH reductase. Methylene blue reduces half-life of methemoglobin from 15-20 hours to 40-90 minutes. We also administered vitamin C 1gm/day and dextrose containing fluids for supplementing NADH/NADPH which is needed for reduction of methemoglobin by NADPH reductase enzyme. The patient's ventilation was supported mechanically for few days. He made an uneventful recovery and was discharged home after seven days.

In our patient, methemoglobinemia had occurred following ingestion of indoxacarb, a pesticide, which was identified due to a high degree of suspicion and treated early with methylene blue. Literature search for methemoglobinemia secondary to indoxacarb did not retrieve any articles. Toxic effects of indoxacarb in humans are not described.[5] Hence, this case report of methemoglobinemia secondary to indoxacarb poisoning.
